# Supplementary material for: Global patterns and climatic controls of forest structural complexity
Source: Nat Commun. 2021 Jan 22;12:519. doi: 10.1038/s41467-020-20767-z (PMC7822964; doi:10.1038/s41467-020-20767-z)
Supplement: Supplementary file 1 — Supplementary Information [file 41467_2020_20767_MOESM1_ESM.pdf]

Global patterns and climatic controls of forest structural complexity - Supplementary information

Martin Ehbrecht*[1], Dominik Seidel[1], Peter Annighöfer[2], Holger Kreft[3,4], Michael Köhler[5], Delphine Clara Zemp[3], Klaus Puettmann[6], Reuben Nilus[7], Fred Babweteera[8,9], Katharina Willim[1], Melissa Stiers[1], Daniel Soto[10], Hans Juergen Boehmer[11,12], Nicholas Fisichelli[13], Michael Burnett[14,15], Glenn Juday[16], Scott L. Stephens[17], Christian Ammer[1,4]

*corresponding author: martin.ehbrecht@forst.uni-goettingen.de

[1]Silviculture and Forest Ecology of the Temperate Zones, University of Göttingen, Büsgenweg 1, 37077 Göttingen, Germany

[2]Forest and Agroforest Systems, Technical University of Munich (TUM), Hans-Carl-von-Carlowitz-Platz 2, 85354 Freising, Germany

[3]Biodiversity, Macroecology and Biogeography, University of Göttingen, Büsgenweg 1, 37077 Göttingen, Germany

[4]Centre of Biodiversity and Sustainable Land Use (CBL), University of Göttingen, Büsgenweg 1, 37077 Göttingen, Germany

[5]Nordwest German Forest Research Institute, Grätzelstr. 2, 37079 Göttingen, Germany

[6]Department of Forest Ecosystems and Society, Oregon State University, Corvallis, OR 97331, USA

[7]Forest Research Centre, Sabah Forestry Department, P.O. Box 1407, 90715 Sandakan, Malaysia

[8]Budongo Conservation Field Station, P.O. Box 362, Masindi, Uganda

[9]Department of Forestry, Biodiversity and Tourism, Makerere University, P.O. Box 7062, Kampala, Uganda

[10]Departmento de Recursos Naurales y Tecnología, Universidad de Aysén, Obispo Vielmo 62, Coyhaique, Chile

[11]School of Geography, Earth Science, and Environment, University of the South Pacific, Laucala Bay, Suva, Fiji

[12]Institute of Geography, University of Jena, Löbdergraben 32, 07743 Jena, Germany

[13]Schoodic Institute at Acadia National Park, P.O. Box 277, Winter Harbor, Maine 04693, USA

[14]Earth Systems Program, Stanford University, 473 Via Ortega, Stanford, CA 94305, USA

[15]The Nature Conservancy, 67-1197 Mamalahoa Hwy., P.O. Box 1056, Kamuela, HI 96743, USA

[16]Department of Natural Resources and Environment, and Institute of Agriculture, Natural Resources and Extension, University of Alaska Fairbanks, P.O. Box 7566180, Fairbanks Alaska 99775, USA

[17]Department of Environmental Science, Policy, and Management, 130 Mulford Hall, University of California, Berkeley, CA, 94720, USA

Supplementary Note 1. Distribution of study sites across the climatic range of sampled biomes

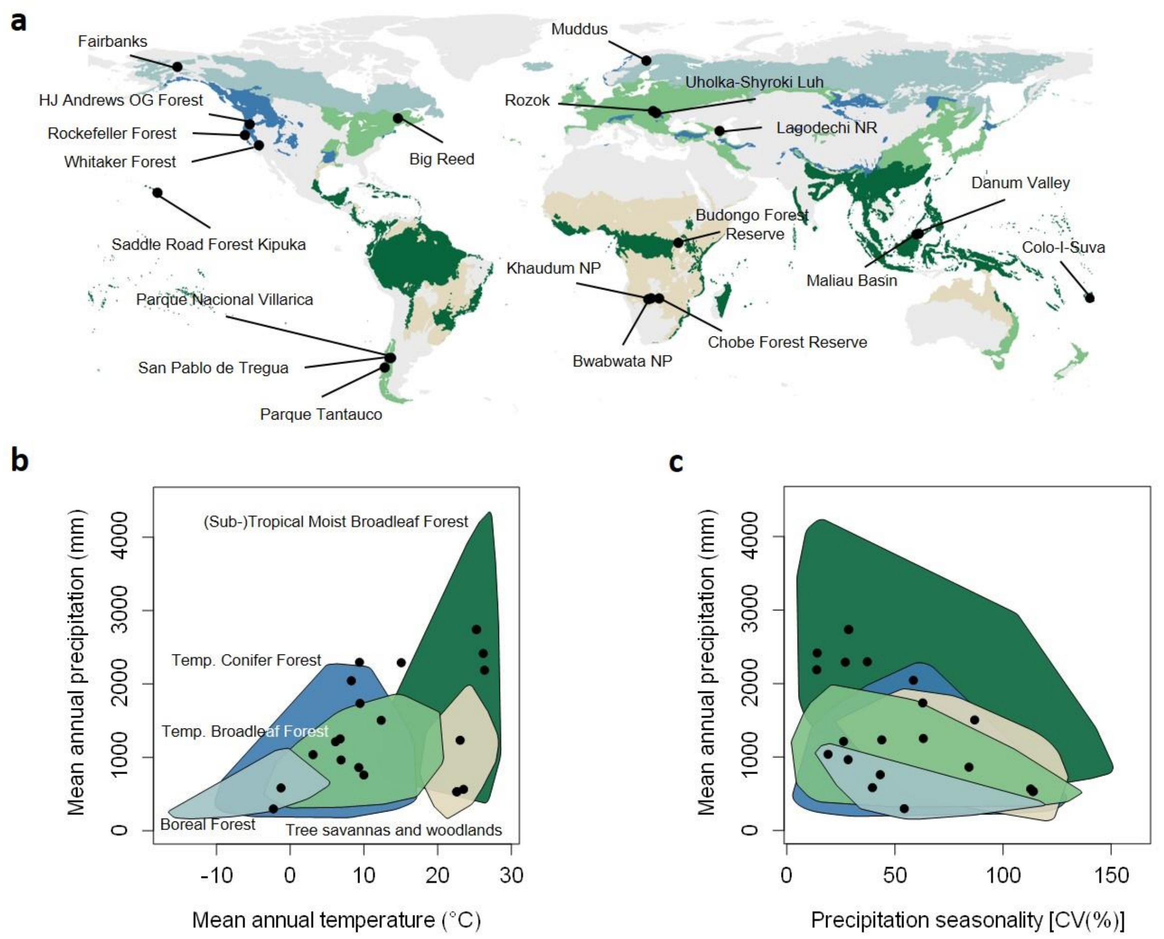

Supplementary Fig. 1. Distribution of study sites across the sampled biomes and climatic range. Biome boundaries in (a) are based on shapefiles published by Olson et al. (2001)[1]. Colored polygons in (b) and (c) represent the climatic range of studied biomes.

The sites used in this study reflected the global climatic range of forest and woodland biomes quite well (Supplementary Fig. 1). Forests with annual precipitation ≤ 300 mm or ≥ 2700 mm and a precipitation seasonality of more than ≥ 113 % or less than ≤ 14 % (coefficient of variation of monthly precipitation) accounted for ~3 % of the forest and woodland points in our global sampling grid, after excluding outliers based on a 95 % kernel density estimation for each biome (see Supplementary Fig. 9). Forest structural complexity is unlikely to linearly continue to increase or decrease far beyond the range we have studied and should saturate at a certain asymptote. We assume SSCI would follow a sigmoid function if sites with higher and lower precipitation and seasonality were included. However, structural complexity may also

decrease with extremely high levels of precipitation due to permanent waterlogging. Still, we measured SSCI values of up to 13.4 in individual plots, suggesting that higher SSCI values in the excess of site-averaged SSCI values are possible within the investigated climatic range.

Supplementary Note 2. Correlations between the Stand Structural Complexity Index (SSCI) and other metrics of forest structural complexity

The Stand Structural Complexity Index (SSCI) used in this study correlates well with other metrics of forest structural complexity (Supplementary Fig. 2, such as the Structural Complexity Index by Zenner & Hibbs (2000)[2], the Gini-Coefficient of tree diameters (see [3]) and the Tree Size Differentiation Index (see [4]).

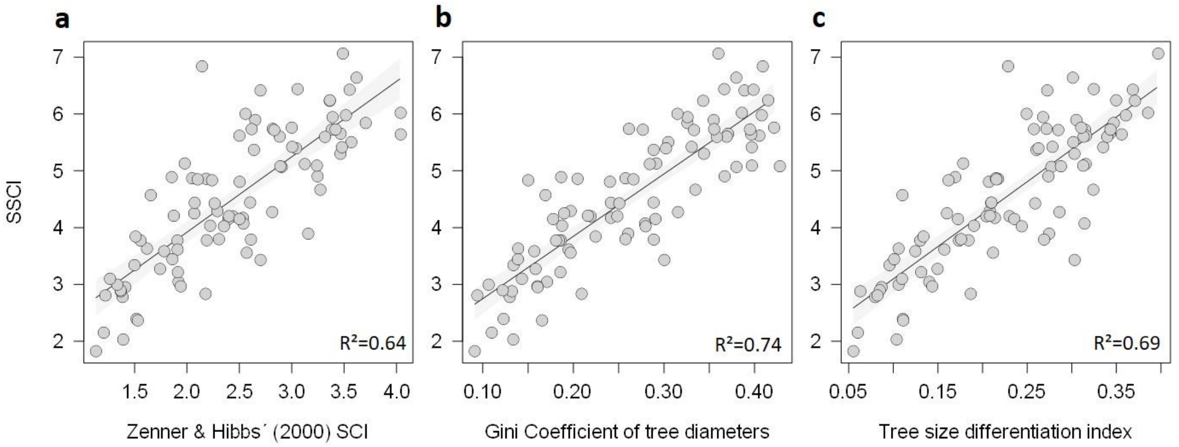

Supplementary Fig. 2. Relationships of SSCI with other metrics of forest structural complexity. Linear regression was used to model relationships between the forest structural complexity index used in this study (SSCI) and (a) the Stand structural complexity index (SCI) by Zenner and Hibbs (2000)[2], (b) the Gini-Coefficient of tree diameters (see[3]), and (c) the tree size differentiation index (see[4]). Data points are based on 91, fully inventoried, stem-mapped forest plots of 1 hectare (n = 91). Each linear regression model was significant at $p < 0.0001$. Forest plots represent differently managed temperate broadleaf and conifer forests of Central Europe (shelterwood system, selectively logged and unmanaged, old-growth). Data points represent mean SSCI-values of five systematically distributed single scans per plot (same sampling design as in this study). Shaded envelopes represent the 95 % confidence intervals of the regression lines. The shown correlations between SSCI and other measures of structural complexity are stronger than in the original publication[5], because they are based on fully inventoried and completely stem-mapped plots (in contrary to the results shown in the original publication, where trees smaller than 7 cm DBH were not accounted for). Additional information on study sites is provided in [5,6]. Data of tree attribute-based metrics of forest structural complexity is published in [6].

The diversity and intermingling of different tree sizes, in conjunction with complementary crown architectures, determine the density and degree of heterogeneity of biomass or foliage

distribution in three-dimensional space and thus the vertical stratification and spatial patterns of canopy space occupation (see Supplementary Fig. 3). SSCI consists of two components, the fractal dimension of cross-sectional polygons and effective number of canopy layers (Effective number of layers, ENL[7]).

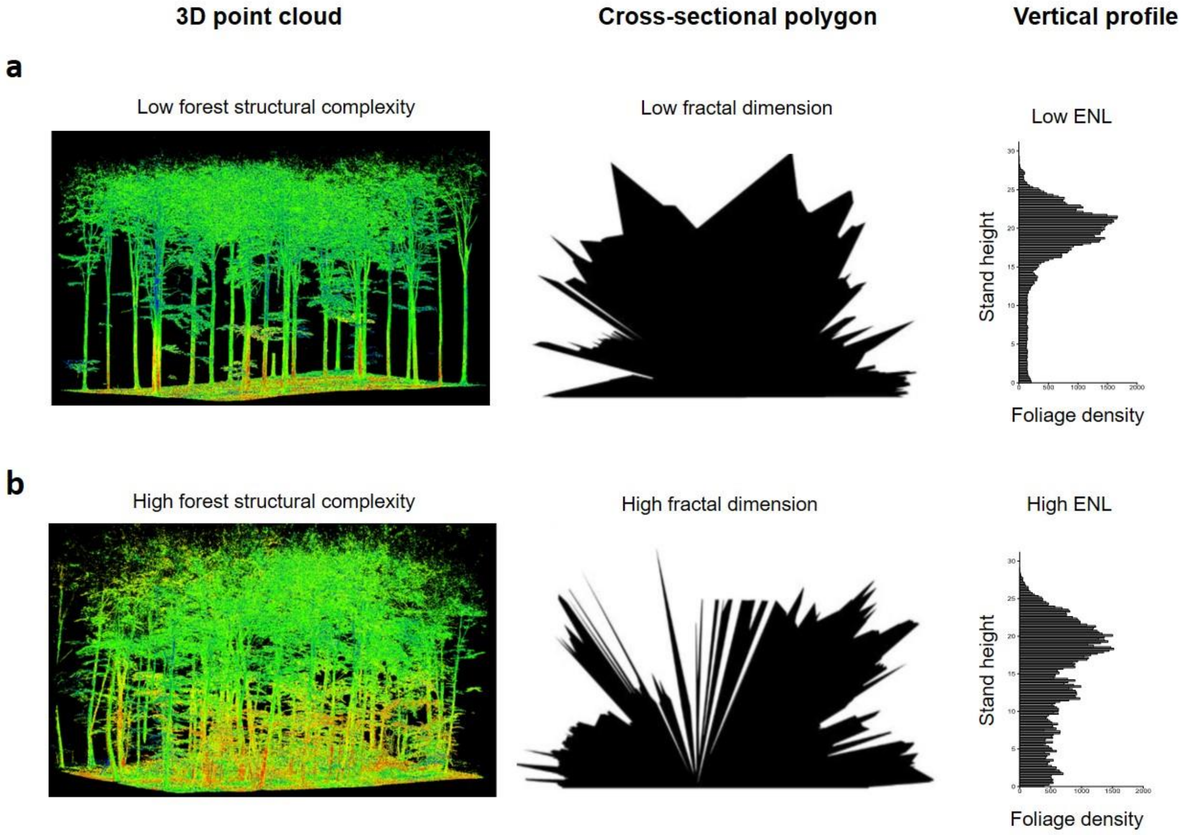

Supplementary Fig. 3. Graphical visualization of three-dimensional point clouds, cross-sectional polygons and vertical foliage profiles of a forest with low (a, SSCI = 4.3) and high structural complexity (b, SSCI = 7.1). Images of three-dimensional point clouds are based on a dataset published in Juchheim et al. (2017)[8]. Images of cross-sectional polygons and vertical profiles are based on a dataset published in Ehbrecht et al. (2017)[5].

The fractal dimension quantifies the shape complexity of cross-sectional polygons (see [5]for details). As such, it quantifies the heterogeneity of foliage or biomass distribution in three-dimensional space (sensu[9]). However, the fractal dimension is a scale-independent measure of structural complexity (see [10]). In order take stand size and vertical stratification into account, the Effective Number of Layers (ENL) is used to scale the fractal dimension values, which quantifies the vertical stratification or layering. Higher tree size diversity and intermingling result in multi-layered canopies and thus higher ENL values (see vertical profiles of foliage density in Supplementary Fig. 3, ENL is in the right profile is higher than in the left).

SSCI is based on single terrestrial laser scans and as such, it is affected by occlusion, because parts of the canopy space are sampled under-proportionally. To test effects of occlusion on ENL, we compared ENL values between point clouds based on single scans and multiple scans with occlusion of less than 1% (based on a voxel size of 20cm side length. Ray tracing was used to determine the share of occluded voxels, around 40 scans on a 40 x 40 m plot, see [7] for details). We found that ENL can be quantified based on single scans with an RMSE of 9.82% (see Fig. 8). The slope of the regression line, however, was not significantly different from 1 (i.e. no significant deviation from the 1:1 line). The cross-sectional polygons are based on the hemispherical view of the scanner, and as such, the fractal dimension is not directly affected by occlusion. Inherent to the way they are being constructed, the cross-sectional polygons solely depend on information that is visible from the scanner´s perspectives. Even though large parts of the upper canopy remain occluded, laser beams still traverse through small gaps in lower- or mid-canopy layers and detect objects in upper canopy layers, which creates spikes in the cross-sectional polygons. A more heterogeneous distribution of foliage or biomass in three-dimensional space thus results in more complex shapes of the cross-sectional polygons and is expressed in higher fractal dimension values.

Supplementary Note 3. Correlations between forest structural complexity and canopy height, basal area or canopy openness

Contrary to other studies[11,12], we did not find significant correlations between climate variables and canopy height or basal area, which are often used as predictors of above-ground biomass[13]. However, we still assume that forest structural complexity could be correlated to these structural attributes, as climatic effects on structural complexity could be mediated by correlations between structural complexity and canopy height, basal area or canopy openness. SSCI followed a humped-shaped curve in relation to canopy height and basal area and decreased with increasing canopy openness (Supplementary Fig. 4). Forests with very high and very low basal area/canopy height are rather characterized by a low structural complexity.

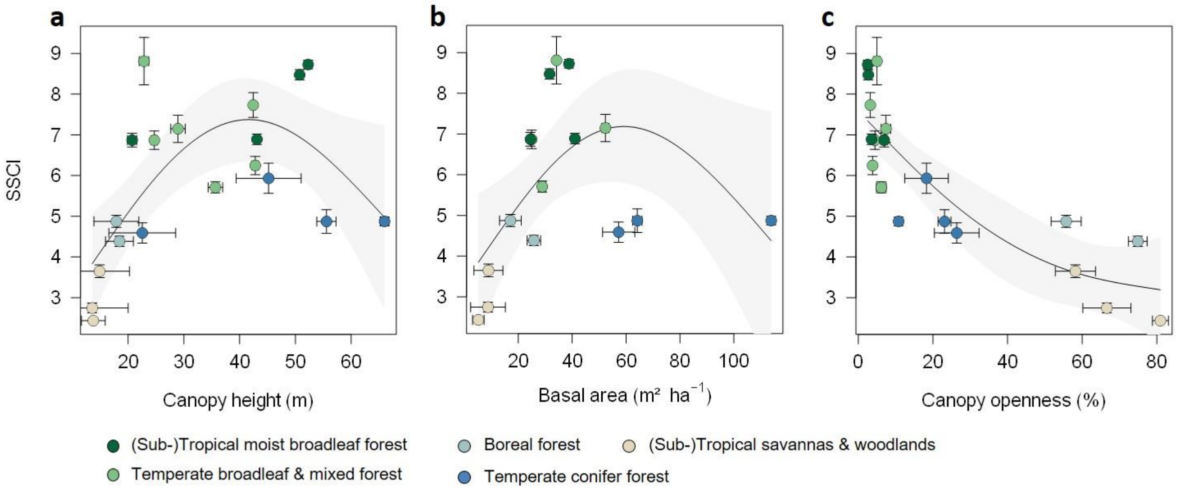

Supplementary Fig. 4. Relationships of canopy height (a), basal area (b) and canopy openness (c) with the stand structural complexity index (SSCI) for the study sites. Fitted curves represent thin-plate regression splines based on generalized additive models. Shaded enveloped represent 95 % confidence intervals of regression splines. Models were significant at $p = 0.02$ (a), $p = 0.0958$ (b) and $p < 0.0001$ (c). Data points represent mean SSCI as well as canopy height, basal area or canopy openness values, respectively, per site. Error bars indicate the standard error of the mean.

Greater canopy height and basal area are both mainly driven by the number of large diameter trees on the plot. An increasing share of large diameter trees per plot may result in suppressed growth or lower survival rates of under- or mid-story trees, due to a high degree of asymmetric competition. Under these conditions, tree size diversity is low and three-dimensional canopy space is less efficiently filled, resulting in a lower structural complexity. A low structural complexity of forests with low basal area and/or low canopy height is due to a low tree size diversity or a low vertical stratification, as larger trees are lacking. However, structural complexity decreased with greater canopy openness, following a non-linear, near negative exponential trend (Supplementary Fig. 2).

Supplementary Note 4. Inter-correlations of climate variables

In our dataset, mean annual precipitation (MAP) and mean annual precipitation minus potential evapotranspiration (MAP – PET), as well as precipitation seasonality (Prec. Seasonality) and MAP – PET were significantly inter-correlated (Pearson`s correlation coefficient, $r = 0.79$ and $r = 0.80$, respectively), and were not used in combination during modelling. All other variable combinations either did not show significant inter-correlation or showed correlation coefficients below the threshold ($r < |0.7|$) (Supplementary Fig. 5).

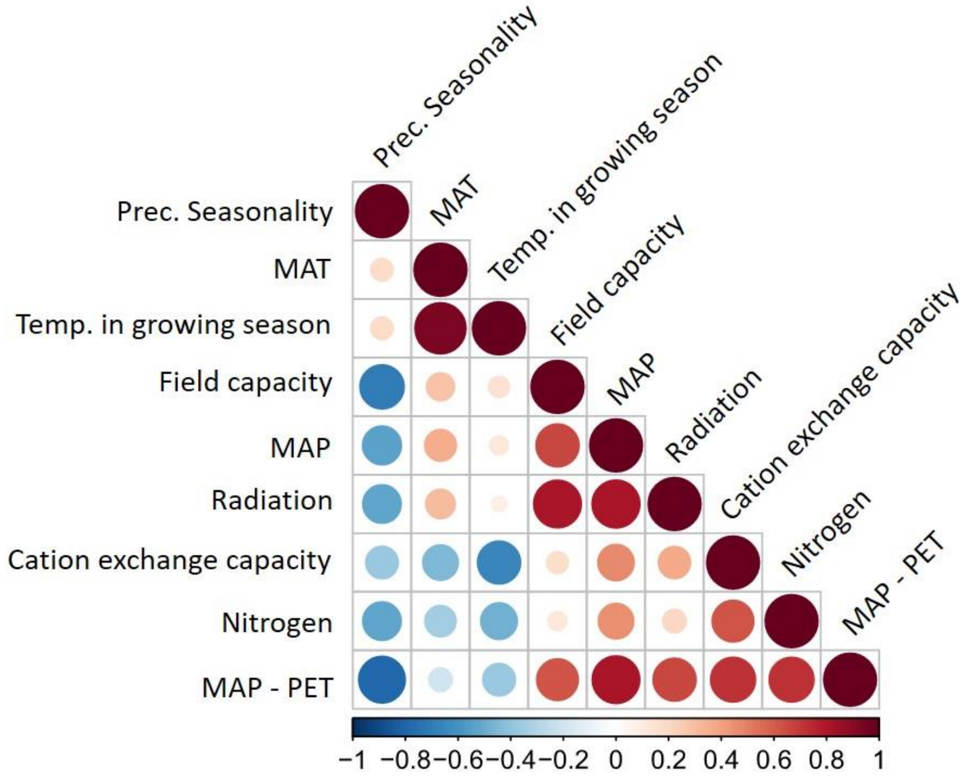

Supplementary Fig. 5. Correlation (r) matrix of climate variables used to check for collinearity.

Supplementary Note 5. Model cross-validation

In order to test the robustness of the model used to predict and map SSCI using mean annual precipitation and precipitation seasonality as predictors, we performed a leave-one-out-cross-validation (LOOCV) using the caret R-package. SSCI of excluded sites was predicted with a mean RMSE of 0.71. Supplementary Table 1 shows the model summaries after excluding single sites or entire biomes. The given summaries describe the model performance (R^2, AIC_c, and RMSE) after excluding the respective sites or biomes.

Supplementary Table 1. Model summaries after excluding single sites or entire biomes from the linear model used estimate the potential structural complexity across the earth`s forest and woodland ecoregions. Each linear regression model was significant at $p < 0.0001$.

**Site excluded**	R^2	AIC_c	**RMSE**
Parque Nacional Villarica (Chile)	0.91	41.69	0.59
Big Reed (USA)	0.89	44.44	0.63
Budongo Forest Reserve (Uganda)	0.90	43.14	0.61
Chobe Forest Reserve (Botsuana)	0.89	43.00	0.61
Bwabwata National Park (Namibia)	0.88	44.38	0.63
Danum Valley (Malaysia)	0.89	44.46	0.63
Fairbanks (USA)	0.89	44.25	0.63
HJ Andrews Old-Growth Forest (USA)	0.90	44.23	0.63
Khaudum National Park (Namibia)	0.88	43.72	0.62
Lagodechi Nature Reserve (Georgia)	0.89	44.43	0.63
Maliau Basin (Malaysia)	0.88	44.45	0.63
Muddus (Sweden)	0.91	40.33	0.57
Parque Tantauco National Park (Chile)	0.90	41.43	0.58
San Pablo de Tregua Nature Reserve (Chile)	0.89	44.06	0.62
Rockefeller Forest (USA)	0.89	44.31	0.63
Rožok (Slovakia)	0.89	44.42	0.63
Saddle Road Forest (USA)	0.92	38.88	0.54
Uholka-Shyrokyi Luh (Ukraine)	0.91	41.64	0.58
Whitaker Forest (USA)	0.90	43.67	0.62
Colo-I-Suva Forest (Fiji)	0.88	44.45	0.63

**Biome excluded**	R^2	AIC_c	**RMSE**
Temperate conifer forest	0.90	37.99	0.62
Temperate broadleaf forest	0.91	32.99	0.59
(Sub-)Tropical moist broadleaf forest	0.89	34.02	0.58
(Sub-)Tropical tree savannas and woodlands	0.82	40.90	0.64
Boreal forest	0.91	39.60	0.58

Supplementary Note 6. Model coefficients and residuals

The normal distribution of model residuals was tested and confirmed using a Shapiro-Wilk test (W = 0.955, p = 0.455) for the model used to predict and map SSCI. Model coefficients are shown in Supplementary Table 2; model residuals are shown in Supplementary Fig. 4.

Supplementary Table 2. Model coefficients of the linear regression model that was used to predict $SSCI_{pot}$ across biomes based on mean annual precipitation (MAP) and precipitation seasonality (Prec. Seasonality)

	Coefficient estimate	Std. error	t	p-value
**Intercept**	6.057558	0.566731	10.689	5.78E-09
**MAP**	0.001319	0.000241	5.473	4.12E-05
**Prec. Seasonality**	-0.033841	0.005521	-6.129	1.11E-05

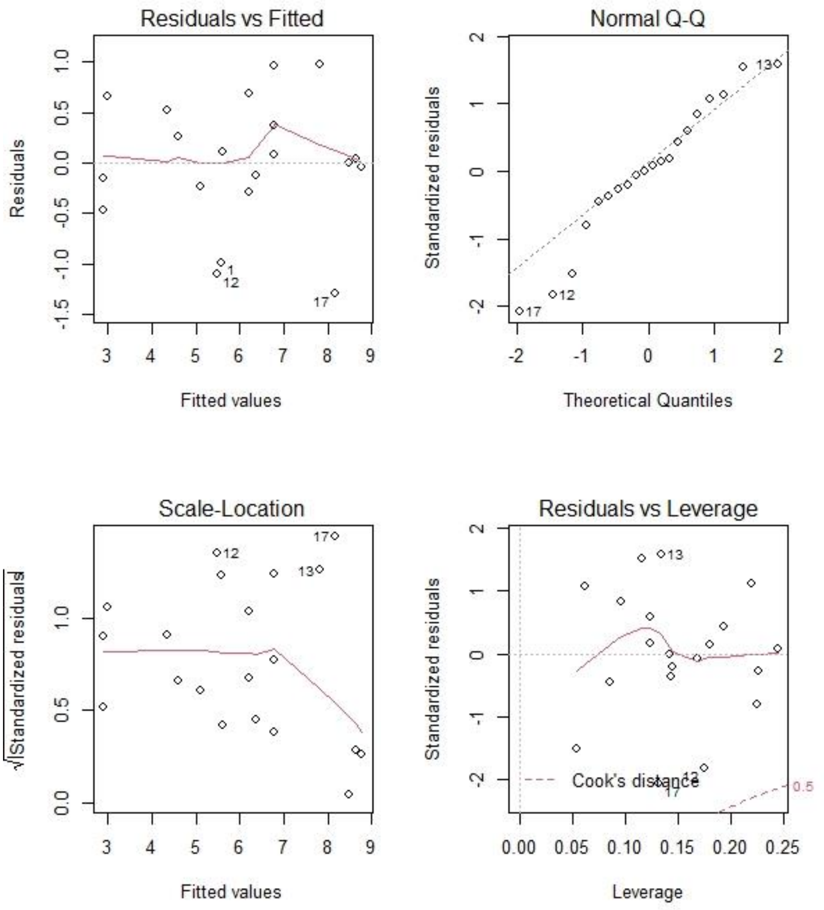

Supplementary Fig. 6. Residual plots of the model that was used to predict $SSCI_{pot}$ across biomes based on mean annual precipitation and precipitation seasonality

Supplementary Note 7. Relations between climate variables and attributes of forest structure

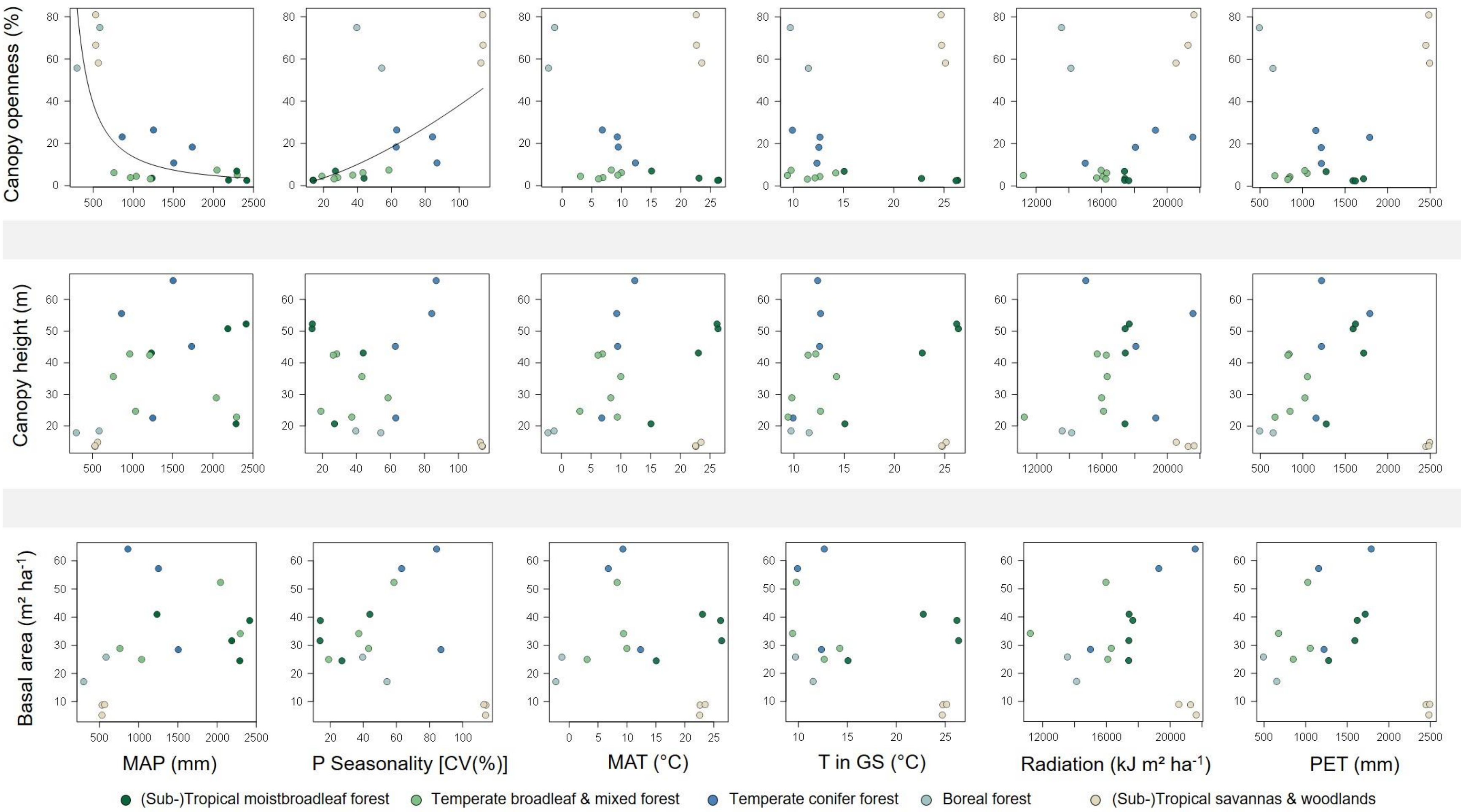

Supplementary Fig. 7. Relations between climate variables and attributes of forest structure. MAP and Prec. seasonality significantly correlated with canopy openness ($p < 0.0001$). We found no significant effects of climate variables on canopy height or basal area.

Supplementary Note 8. Global sampling grid and climatic ranges of sampled biomes

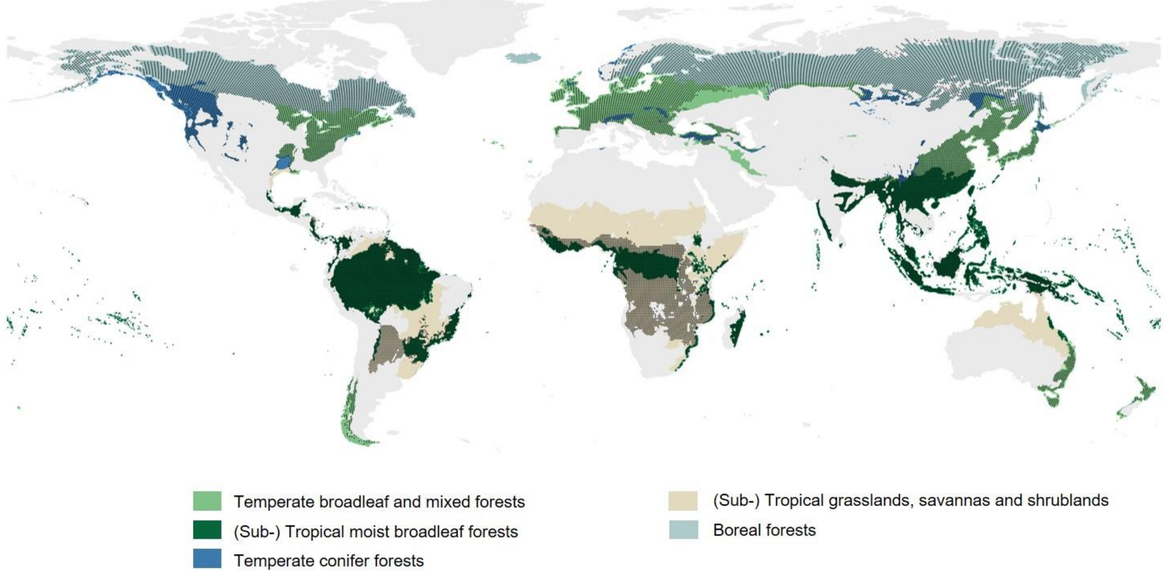

Supplementary Fig. 8. Global sampling grid across the sampled biomes[1]. Distance between sample points is ~50km across latitudes. The sampling grid was confined to ecoregions that were classified as forest, woodland, taiga, chaco, yungas, várzea, or campinarana. Large areas in the (sub-) tropical grasslands, savannas and shrublands biome were not classified as such and were hence not sampled.

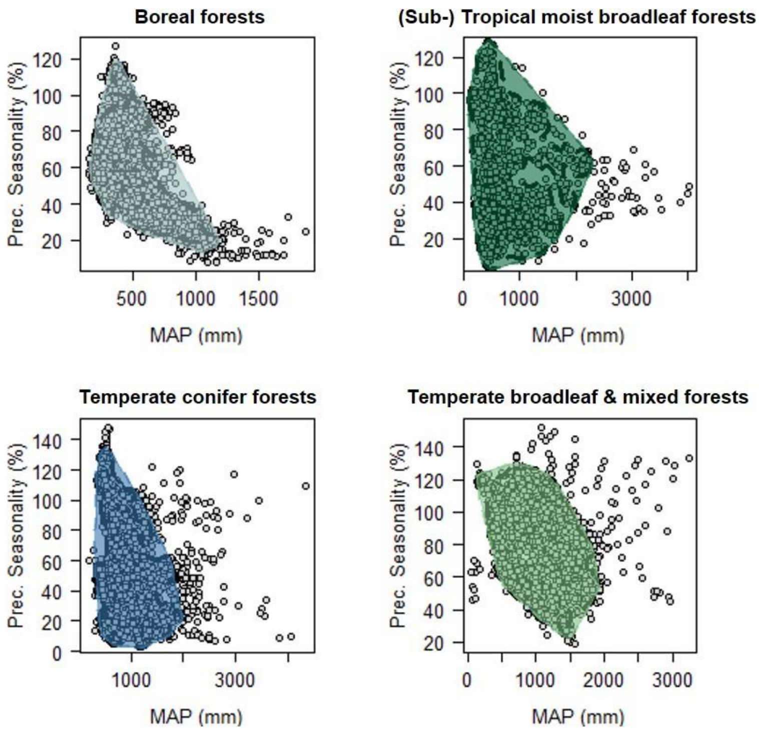

Supplementary Fig. 9. Climatic ranges of sampled biomes. Points represent sample points of the global sampling grid. Dashed lines include the 95 % of sample points that were used to model $SSCI_{pot}$ within biomes, based on a 95 % kernel density estimation. Sample points outside the dashed line were considered outliers and were rejected during analyses. Coloured polygons are convex hulls around the 95 % kernel density used for visualizing the climatic range of the respective biome.

Supplementary Note 9. Relative changes in mean annual precipitation and precipitation seasonality by 2070

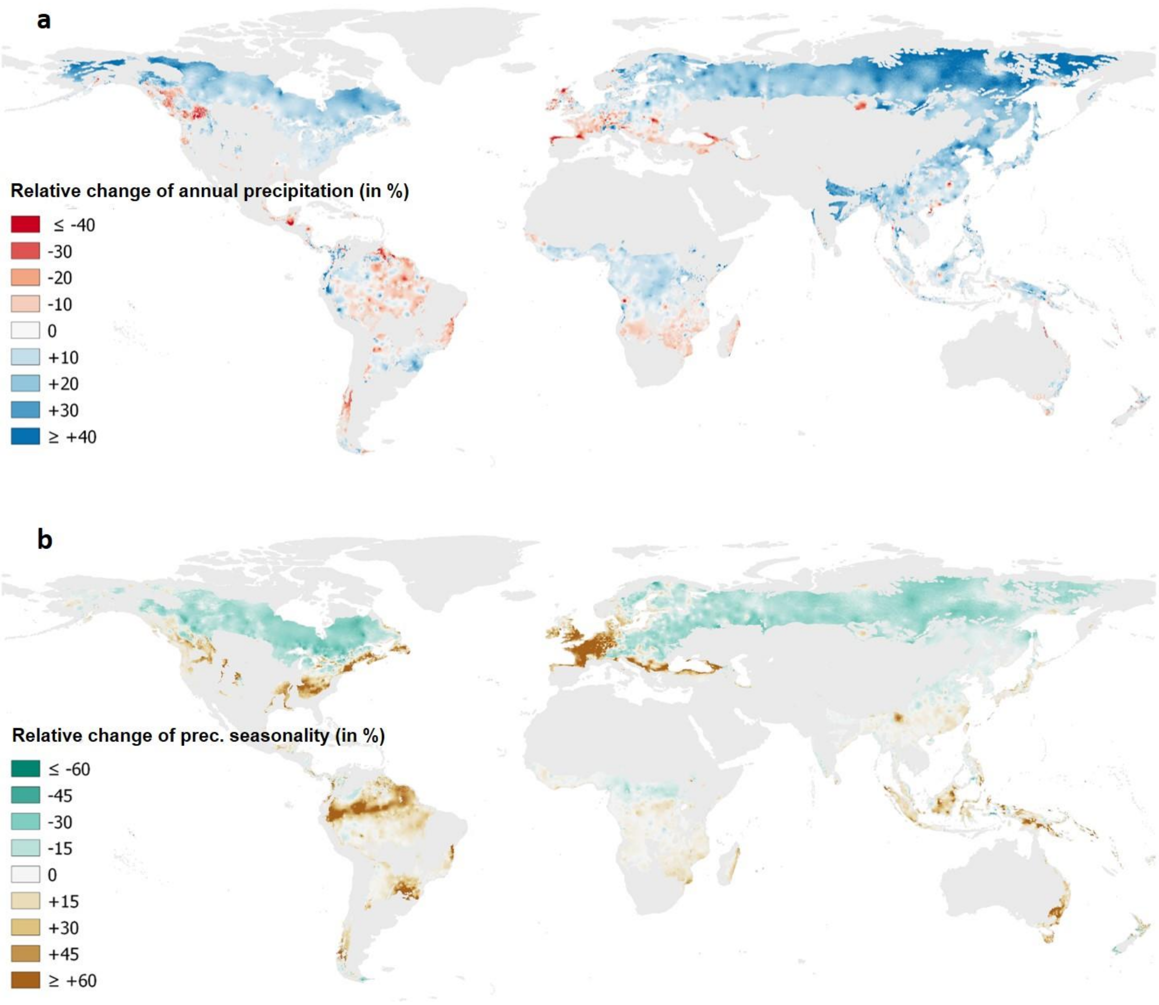

Supplementary Fig. 10. Relative changes in mean annual precipitation (a) and precipitation seasonality (b) under a RCP8.5 emissions scenario. Projections are based on 17 different climate models that were used in the 5th IPCC report[14] within the frame of the Coupled Model Intercomparisons Project (CMIP5)[15]. Maps are based on the WorldClim dataset[16] and show average change across the 17 climate models.

Supplementary References

1. Olson, D. M. et al. Terrestrial Ecoregions of the World: A New Map of Life on EarthA new global map of terrestrial ecoregions provides an innovative tool for conserving biodiversity. BioScience **51**, 933–938 (2001).

2. Zenner, E. K. & Hibbs, D. E. A new method for modeling the heterogeneity of forest structure. For. Ecol. Manag. **129**, 75–87 (2000).

3. Lexerød, N. L. & Eid, T. An evaluation of different diameter diversity indices based on criteria related to forest management planning. For. Ecol. Manag. **222**, 17–28 (2006).
4. Pretzsch, H. Forest Dynamics, Growth, and Yield. in Forest Dynamics, Growth and Yield: From Measurement to Model (ed. Pretzsch, H.) 1–39 (Springer, 2009).
5. Ehbrecht, M., Schall, P., Ammer, C. & Seidel, D. Quantifying stand structural complexity and its relationship with forest management, tree species diversity and microclimate. Agric. For. Meteorol. **242**, 1–9 (2017).
6. Schall, P., Schulze, E.-D., Fischer, M., Ayasse, M. & Ammer, C. Relations between forest management, stand structure and productivity across different types of Central European forests. Basic Appl. Ecol. **32**, 39–52 (2018).
7. Ehbrecht, M., Schall, P., Juchheim, J., Ammer, C. & Seidel, D. Effective number of layers: A new measure for quantifying three-dimensional stand structure based on sampling with terrestrial LiDAR. For. Ecol. Manag. **380**, 212–223 (2016).
8. Juchheim, J., Ammer, C., Schall, P. & Seidel, D. Canopy space filling rather than conventional measures of structural diversity explains productivity of beech stands. For. Ecol. Manag. **395**, 19–26 (2017).
9. Gough, C. M., Atkins, J. W., Fahey, R. T., Hardiman, B. S. & LaRue, E. A. Community and structural constraints on the complexity of eastern North American forests. Glob. Ecol. Biogeogr. **n/a**, (2020).
10. Mandelbrot, B. B. Stochastic models for the Earth's relief, the shape and the fractal dimension of the coastlines, and the number-area rule for islands. Proc. Natl. Acad. Sci. **72**, 3825–3828 (1975).
11. Klein, T., Randin, C. & Körner, C. Water availability predicts forest canopy height at the global scale. Ecol. Lett. **18**, 1311–1320 (2015).

12. Tao, S., Guo, Q., Li, C., Wang, Z. & Fang, J. Global patterns and determinants of forest canopy height. Ecology **97**, 3265–3270 (2016).

13. Slik, J. W. F. et al. Environmental correlates of tree biomass, basal area, wood specific gravity and stem density gradients in Borneo's tropical forests. Glob. Ecol. Biogeogr. **19**, 50–60 (2010).

14. IPCC. Climate Change 2014: Synthesis Report. Contribution of Working Groups I, II and III to the Fifth Assessment Report of the Intergovernmental Panel on Climate Change [Core Writing Team, R.K. Pachauri and L.A. Meyer (Eds.)]. IPCC, Geneva, Switzerland. (2014).

15. Taylor, K. E., Stouffer, R. J. & Meehl, G. A. An Overview of CMIP5 and the Experiment Design. Bull. Am. Meteorol. Soc. **93**, 485–498 (2011).

16. Fick, S. E. & Hijmans, R. J. WorldClim 2: new 1-km spatial resolution climate surfaces for global land areas. Int. J. Climatol. **37**, 4302–4315 (2017).
